# COVID-19 pandemic and violence: rising risks and decreasing urgent care-seeking for sexual assault and domestic violence survivors

**DOI:** 10.1186/s12916-020-01897-z

**Published:** 2021-02-05

**Authors:** Katherine A. Muldoon, Kathryn M. Denize, Robert Talarico, Deshayne B. Fell, Agnes Sobiesiak, Melissa Heimerl, Kari Sampsel

**Affiliations:** 1 Ottawa Hospital Research Institute, 501 Smyth Road, Ottawa, Ontario K1H-8L6 Canada; 2 School of Epidemiology and Public Health, University of Ottawa, Ottawa, Ontario Canada; 3 Children’s Hospital of Eastern Ontario Research Institute, Ottawa, Ontario Canada; 4 Ottawa Victims Services, Ottawa, Ontario Canada; 5 Faculty of Medicine, Department of Emergency Medicine, The Ottawa Hospital and University of Ottawa, Ottawa, Ontario Canada

**Keywords:** COVID-19, Violence, Sexual assault, Intimate partner violence, Emergency department, Gender-based violence, Epidemiology, Emotional abuse

## Abstract

**Background:**

There is little information on care-seeking patterns for sexual assault and domestic violence during the COVID-19 pandemic. The objective of this study was to examine the changes in emergency department (ED) admissions for sexual assault and domestic violence since the COVID-19 pandemic was declared.

**Methods:**

Observational ED admissions data from The Ottawa Hospital were analyzed from March 4 to May 5 (62 days) in 2020 (COVID-19 period) and compared to the same period in 2018 (pre-COVID-19). Total and mean weekly admissions were calculated for all-cause ED admissions and for sexual and domestic violence cases. A Poisson regression (without offset term) was used to calculate the weekly case count ratio and 95% confidence intervals (CI) between the two time periods. Case characteristics were compared using chi-square tests, and percent differences were calculated.

**Results:**

Compared to pre-COVID-19, total ED admissions dropped by 1111.22 cases per week (32.9% reduction), and the Sexual Assault and Domestic Violence Program cases dropped 4.66 cases per week. The weekly case count ratio for sexual assault cases was 0.47 (95% CI 0.79–0.27), equivalent of 53.49% reduction in cases, and 0.52 (95% CI 0.93–0.29), equivalent to a 48.45% reduction in physical assault cases. The characteristics of presenting cases were similar by age (median 25 years), sex (88.57% female), assault type (57.14% sexual assault, 48.57% physical assault), and location (31.43% patient’s home, 40.00% assailant’s home). There was a significant increase in psychological abuse (11.69% vs 28.57%) and assaults occurring outdoors (5.19% vs 22.86%).

**Conclusion:**

This study found a decrease in ED admissions for sexual assault and domestic violence during COVID-19, despite societal conditions that elevate risk of violence. Trends in care-seeking and assault patterns will require ongoing monitoring to inform the provision of optimal support for individuals experiencing violence, particularly as countries begin to re-open or lock-down again.

**Supplementary Information:**

The online version contains supplementary material available at 10.1186/s12916-020-01897-z.

## Background

The extreme stress, uncertainty, and fear that is accompanied by emergencies and disasters, coupled with social isolation and movement restrictions, have raised concern for increases in gender-based violence [1–3]. On March 11, 2020, the World Health Organization declared the novel coronavirus (SARS-CoV-2), or COVID-19, a global pandemic and began efforts to contain the spread of disease through hand hygiene, personal protective equipment, and limited contact with others through physical distancing. On March 27, 2020, the United Nations issued a warning statement that domestic violence may rise due to the restrictive measures implemented to control COVID-19 and called on governments to increase efforts to address the rising risks of violence [4]. To date, the majority of literature on COVID-19 and violence has been released through commentaries, organizational reports, and news sources [5–10]. There is limited empirical evidence on care-seeking for sexual assault and domestic violence [11–13], particularly with data that can be monitored over time to investigate how the patterns of violence change throughout the COVID-19 pandemic.

Evidence from previous pandemics and emergencies [14–17] has identified that the most common factors that increase the risk for violence include economic insecurity, poverty-related stress, job loss or reduced working hours, quarantine, and social isolation. Previous pandemics that required quarantine (e.g., severe acute respiratory syndrome (SARS), influenza A virus subtype H1N1) found that isolation can result in psychological distress, loneliness, depression, stress, post-traumatic stress disorder, anger, sleep disorders, and problematic substance use [18], all factors that increase risk of violence [19–21]. A population-based survey in the USA found that the prevalence of self-reported depression symptoms was 3-fold higher during the first month of COVID-19 (March 31 to April 13, 2020) compared to a time-matched cohort from 2017 [22]. A representative survey administered in April 2020 of 4600 Canadians found that 10% of women and 6% of men were extremely concerned about the possibility of violence in their homes since the COVID-19 crisis began [23]. Importantly, evidence of increased violence may not surface immediately, making it difficult to assess its burden and how to prevent violence and reduce risks.

The COVID-19 pandemic has resulted in changes in service provision from shelters, support hotlines, community-based agencies, and emergency department (ED) [24]; however, the patterns have varied between settings. In the early days of the pandemic (March 31 to April 27, 2020), the National Syndromic Surveillance Program in the USA documented that all-cause ED admissions were 42% lower than time-matched estimates from the year before [25]. Several studies identified concerning patterns of reduced ED admissions for conditions requiring urgent care including heart failure [26, 27] and appendicitis [28]. Studies of assault-based injuries from ED and surgical trauma units have found mixed results. Some have seen no differences in violence-related trauma admission  [13], others found that assault-based trauma cases fell but self-harm increased [12], others found an overall decrease in cases of intimate partner violence; however, the injuries were more severe [11]. Importantly, survivors may not present for fear of contracting COVID-19 or may feel that their issues are not important enough to seek help and may not want to “burden” an already overwhelmed health system.

Some community agencies and service providers have experienced spikes in the volume [29, 30], while others have seen decreases since physical distancing policies went into effect [31]. Most, if not all, community agencies and service providers have introduced modifications, including the closure of physical offices, further reducing options for survivors to access support on a walk-in basis. Survivors of violence or those at risk of violence may be unaware of what services remain open.

While many agencies have begun to offer services remotely [32], in-person visits are critical for sexual assault and domestic violence cases involving treatment for physical injuries (e.g., fractures, lacerations, strangulation), HIV post-exposure prophylaxis [33], or forensic evidence collection (Sexual Assault Evidence Kit, also known as the “rape kit”), for those wishing to pursue legal action [34]. The increased reliance on remote care may also pose a risk to those living with controlling or abusive partners, as they may not have a safe space for a remote consultation with a service provider.

The objectives of this study were to evaluate the impact of the COVID-19 pandemic on ED admissions for sexual assault and domestic violence cases compared to a time-matched control group from 2018 to (1) examine changes in ED visits for sexual assault and domestic violence cases, and (2) compare sociodemographic, clinical, and assault-related characteristics of the presenting cases between the two time periods.

## Methods

### Study setting

This study was conducted in Ottawa, Ontario, the fourth largest city in Canada with a census metropolitan population of 1.3 million. At the time of the study, there were 2650 COVID-19 cases in Ottawa (2240 recovered), 40,161 cases in Ontario (36,381 recovered), and 119,451 cases in Canada (112,709 recovered) [35]. Data for this study come from two EDs at The Ottawa Hospital, a multi-campus tertiary-level care hospital, with a specialized Sexual Assault and Domestic Violence Program based within the EDs. The program is one of 36 specialized Sexual Assault and Domestic Violence Treatment Centers across the province of Ontario and is the only location in the region that collects forensic evidence and administers Sexual Assault Evidence Kits. Admitted patients are triaged to the Sexual Assault and Domestic Violence Program if they self-disclose or are identified by staff as experiencing sexual assault or domestic violence. The Sexual Assault and Domestic Violence Program offers 24/7 clinical care to individuals 16 years of age or older, including all women, men, transgender, and gender non-binary individuals. Emergency care includes injury assessment, documentation, and treatment; sexually transmitted infection (STI) testing; emergency contraception and pregnancy testing; HIV post-exposure prophylaxis; immunization for hepatitis B, hepatitis A, and human papillomavirus; forensic photo documentation of injuries; collection/storage of the Sexual Assault Evidence Kit [34]; crisis counseling; and risk/threat assessment and safety planning. There were no reductions or changes to program staffing, procedures, or services since the COVID-19 pandemic began, except for an increase in personal protective equipment (e.g., universal masking) as part of the hospital-wide policy.

### Study design

This was an observational study of changes in ED admissions for sexual assault and domestic violence seen during the COVID-19 period compared to a time-matched control group from 2018. In Ontario, the provincial government declared a state of emergency in March 2020, to reduce the spread of COVID-19. This declaration allowed the government to shut down most public establishments (e.g., schools, childcare centers, libraries, recreational centers, restaurants, theaters, and concert venues) and most workplaces transitioned to remote work, where possible. By investigating the ED admissions data, a drastic decrease in admissions began on 4 March 2020, which we chose as the cutoff date. Data from March 4 to May 5, 2020, were categorized as the COVID-19 period (62 days) and compared to March 4 to May 5 in 2018 (pre-COVID-19 period). Case data came from a detailed registry of all patients seen in the Sexual Assault and Domestic Violence Program. ED patients are triaged to the program if they self-disclose or are clinically assessed as having experienced sexual assault, domestically violent event, or a physical assault by an intimate partner or other person. General ED admissions data came from the Health Records department at The Ottawa Hospital.

All cases were reviewed in detail, and all assault, clinical, and demographic data were extracted by research assistants (KD, AS). To ensure inter-rater reliability, 10% of the charts were randomly selected and evaluated by the Medical Director of the Sexual Assault and Domestic Violence Program (KS). The kappa statistic was 0.94 (95% CI 0.88–0.97) indicating strong inter-rater reliability.

### Outcomes

Sexual assaults included any type of assault of a sexual nature, including oral, vaginal, or anal penetration; groping; or any unwanted sexual touching of body parts. Physical assaults included assault by bodily force, strangulation, or assault with a weapon/object.

### Covariables

Demographic characteristics included age, measured both continuously and categorized at 24 years of age; gender/sex (female vs male/transgender/non-binary); method of arrival (ambulance vs walk-in); and police involvement. Mental health conditions could be self-reported or previously documented in the medical charts and included depression, anxiety, and substance use or dependency. Information on the assailant(s) included whether the assailant was an intimate partner, unknown assailant, or multiple assailants. Assault-related information included psychological abuse (e.g., humiliating, threatening, controlling behavior), the use of weapons, strangulation, and location of assault (patient’s home, assailant’s home, outdoors, other). Clinical charts were reviewed for any documentation of visible injuries including lacerations, fractures, or contusions.

Health- and justice-related service provision included uptake of HIV post-exposure prophylaxis (PEP) and administration of the Sexual Assault Evidence Kit or forensic photography. PEP is offered to patients who arrive within 72 h of the assault and are considered moderate-to-high risk defined as follows: being assaulted by an assailant with a known HIV+ status, or an assailant with high risk of HIV (e.g., history of injection drug use, men who has sex with men, migrating from an HIV endemic area) or if there was a known or unknown exchange of body fluids via vaginal and/or anal penetration. PEP was categorized as a three-level variable: non-eligible, eligible but not started, and eligible and started.

Patients are eligible to complete a Sexual Assault Evidence Kit if they meet the following criteria: presenting 12 days following a vaginal/penile assault, 7 days following a digital-vaginal assault, 3 days following a penile/digital anal assault, and 24 h following a penile-oral assault. The Sexual Assault Evidence Kit was categorized as a four-level variable: non-eligible, eligible but not collected, collected and released to police, and collected but not released to police. Patients are eligible for forensic photography if they have visible injuries (e.g., bruises, lacerations, fractures), and the categories were as follows: non-eligible, eligible but not completed, and eligible and completed.

### Analyses

All analyses were conducted using SAS 9.4 [36]. Descriptive statistics include frequencies and percentages for categorical variables. Continuous variables were summarized using median and interquartile range (IQR).

Total admissions and mean weekly admissions were calculated for the 2018 pre-COVID-19 period and the 2020 COVID-19 period using all-cause ED admissions, and specifically admissions to the Sexual Assault and Domestic Violence Program. The absolute difference was calculated as the differences between the two periods. The relative percent change was calculated as the difference between the two periods divided by the 2018 pre-COVID-19 totals and expressed as a percentage. Analyses comparing multiple years of data can be found in Additional file 1: Tables S1 and S2).

A Poisson regression (without offset term) was used to calculate the weekly case count ratio and 95% confidence intervals (CI) between the two periods for the number of patients seen in Sexual Assault and Domestic Violence Program patients. Sexual assault cases and physical assault cases were analyzed individually. Percentage relative reduction was calculated as 1/weekly case count ratio and expressed as a percentage.

The characteristics of the sexual assault and domestic violence patients were compared between the two periods using chi-square tests for categorical variables, and percent difference and 95% CI were calculated. Wilcoxon’s ranked sum test was calculated for continuous variables. All cases were included, and every record was analyzed (i.e., no missing data).

This study was approved by The Ottawa Health Sciences Network Research Ethics Board (Protocol number: 20170390-01H).

## Results

Table 1 presents the differences in the patient volume in the 2018 pre-COVID-19 period and 2020 COVID-19 period. During the 2020 COVID-19 period, the ED saw a 32.93% reduction in patient volume, equating to 1111.22 fewer patients per week. The Sexual Assault and Domestic Violence Program saw a 55.84% decrease in total patient volume, with 4.66 fewer patients per week presenting for care. There was a 56.52% decrease in sexual assault cases and 48.48% in physical assault cases presenting for care.

**Table 1:** Comparison of ED admissions for sexual assault and domestic violence between March 4 and May 5 (*n* = 62 days) in 2018 (pre-COVID-19 period) and 2020 (COVID-19 period)

	2018	2020	Absolute difference	Percent relative reduction (%)
ED admissions				
Total ED admissions	30,371	20,370	10,001	32.93
Mean weekly ED admissions	3374.56	2263.33	1111.22	32.93
Sexual Assault and Domestic Violence Program patients				
Total patients	77	34	43	55.84
Mean weekly patients	8.55	3.89	4.66	54.50
Case rate per 10,000 ED admissions	25.35	17.18	8.17	32.23
Sexual assault cases^1^				
Total cases	46	20	26	56.52
Mean weekly cases	5.11	2.22	2.89	56.56
Case rate per 10,000 ED admissions	15.15	9.82	5.33	35.18
Physical assault cases				
Total cases	33	17	16	48.48
Mean weekly cases	3.67	1.88	1.79	48.77
Case rate per 10,000 ED admissions	10.87	8.35	2.52	23.18

^1^The total sexual assault and physical assault do not sum to 100% as patients can experience both sexual and physical assaults

Table 2 presents the Poisson regression modeling. In the 2020 COVID-19 period, the weekly case count ratio for the Sexual Assault and Domestic Violence Program was 0.45 (95% CI 0.68–0.30) compared to 2018 pre-COVID-19 period, or a 54.55% reduction in weekly counts. Sexual assault cases were 0.47 (95% CI 0.79–0.27) or a 53.49% reduction in weekly cases presenting for care, and physical assault cases were 0.52 (95% CI 0.93–0.29) or a 48.45% reduction in weekly cases presenting for care.

**Table 2:** Poisson regression modeling the weekly case count ratio in 2020 (COVID-19 period) to time-matched 2018 pre-COVID-19 comparison group

Time frame: March 4 to May 5	Weekly case count ratio (95% CI)^1^	Percentage relative reduction^2^ (%)	*p* value
Sexual Assault and Domestic Violence Program patients			
2020	0.45 (0.68–0.30)	54.55 (31.97–69.51)	0.001
2018	(Ref)		
Sexual assault			
2020	0.47 (0.79–0.27)	53.49 (20.63–72.60)	0.002
2018	(Ref)		
Physical assault			
2020	0.52 (0.93–0.29)	48.45 (7.41–71.35)	0.026
2018	(Ref)		

^1^Weekly case count ratio calculated with a Poisson regression without offset term comparing weekly counts in 2020 to 2018

^2^(1/weekly case count ratio) × 100

Table 3 displays the proportional differences in the composition of the patients admitted to the Sexual Assault and Domestic Violence Program in the 2020 COVID-19 period compared to the 2018 pre-COVID-19 period. Although there were half as many patients (77 vs 35), the characteristics of the presenting cases were similar. The median age was 25 years (IQR 20–31), and 88.57% were female. Among all patients, 57.14% experienced a sexual assault and 48.57% experienced a physical assault. The most common assailant was an intimate partner (51.43%), and the most common location of assault was the assailant’s home (40.00%) and the patient’s home (31.43%).

**Table 3:** Changes in Sexual Assault and Domestic Violence Program patient characteristics in March 4 to May 5, 2018 and 2020

Variables	2018*, N* = 77, *n* (%)	2020, *N* = 35, *n* (%)	Percent difference^1^ (95% CI)	*p* value
**Demographic characteristics**				
Age (years, median, IQR)	27 (20–34)	25 (20–31)		0.913
Under 24 years	40 (51.95)	19 (54.29)	2.30 (− 17.30, 21.90)	0.818
Female vs male/trans^2^	67 (87.01)	31 (88.57)	1.50 (− 11.30, 14.30)	0.143
Arrived by ambulance (vs walk-in)	18 (23.38)	12 (34.29)	10.90 (− 7.10, 28.90)	0.227
Police involvement	32 (41.56)	17 (48.57)	7.00 (− 12.50, 26.50)	0.488
**Current mental health conditions**				
Depression	29 (37.66)	17 (48.57)	10.90 (− 8.50, 30.30)	0.277
Anxiety	26 (33.77)	11 (31.43)	− 2.30 (− 20.70, 16.10)	0.807
Substance use or dependency	9 (11.69)	8 (22.86)	11.20 (− 4.10, 26.50)	0.127
**Assault characteristics**				
Sexual assault	46 (59.74)	20 (57.14)	− 2.60 (− 22.00, 16.80)	0.796
Physical assault	33 (42.86)	17 (48.57)	5.70 (− 13.90, 25.30)	0.573
Psychological abuse	9 (11.69)	10 (28.57)	16.90 (8.00, 33.00)	0.027
Intimate partner assailant	33 (42.86)	18 (51.43)	8.60 (− 11.00, 28.20)	0.399
Unknown assailant	23 (29.87)	10 (28.57)	− 1.30 (− 19.10, 16.50)	0.889
Multiple assailants	5 (6.49)	< 5	–	–
Strangulation	7 (9.09)	7 (20.00)	10.90 (− 3.40, 25.20)	0.106
Assault with weapon	11 (14.29)	< 5	–	–
Visible injuries^3^	36 (46.75)	17 (48.57)	1.80 (− 17.80, 21.40)	0.858
Most common location of assault^4^				
Patient’s home	26 (33.77)	11 (31.43)	− 2.30 (− 20.70, 16.10)	0.807
Assailant’s home	25 (32.47)	14 (40.00)	7.60 (− 11.40, 26.60)	0.438
Outdoors	4 (5.19)	8 (22.86)	17.60 (3.30, 31.90)	0.005
Other^5^	26 (33.77)	10 (28.57)	− 5.20 (− 23.20, 12.80)	0.585
**Health- and justice-related service provision**				
Post-exposure prophylaxis				
Non-eligible	52 (67.53)	22 (62.86)	− 4.70 (− 23.50, 14.10)	0.640
Eligible, not started	12 (15.58)	8 (22.86)	7.30 (− 8.40, 23.00)	
Eligible, started	13 (16.88)	5 (14.29)	− 2.60 (− 16.70, 11.50)	
Sexual Assault Evidence Kit				
Non-eligible	28 (36.36)	18 (51.43)	15.10 (− 4.30, 34.50)	0.192
Eligible, not collected	28 (36.36)	7 (20.00)	− 16.30 (− 33.20, 0.60)	
Collected, released to police	8 (10.39)	6 (27.14)	16.80 (− 7.10, 20.70)	
Collected, not released to police	13 (16.88)	4 (11.43)	− 5.40 (− 18.70, 7.90)	
Forensic photography				
Non-eligible	41 (53.25)	17 (48.57)	− 4.70 (− 24.30, 14.90)	0.883
Eligible, not completed	19 (24.68)	10 (28.57)	3.90 (− 13.60, 21.40)	
Eligible, completed	17 (22.08)	8 (22.86)	0.80 (− 15.60, 17.20)	

^1^Percent differences only calculated for categorical variables. Continuous variables evaluated by Wilcoxon’s rank sum test

^2^Due to small cell sizes, male and trans/non-binary people are combined

^3^Visible injuries can include lacerations, fractures, and contusions

^4^Most common location of assault—locations do not sum to 100% as assaults can occur in multiple places

^5^Other location includes dorm room, friends’ home, shelter, do not know, and other

A statistically significant increase of 16.90% (95% CI 8.00 to 33.00) was identified in the proportion of cases involving psychological abuse and an increase of 17.60% (95% CI 3.30–31.90) where the assault occurred outdoors. There were no differences in the proportion of patients starting PEP, completing the Sexual Assault Evidence Kit, or forensic photography.

## Discussion

This study has found a drastic drop in the number of patients presenting for care despite the global concern for increases in violence during COVID-19. In the first 2 months of the COVID-19 pandemic, total ED admissions at The Ottawa Hospitals dropped by over 10,000 patients (32.93% reduction) compared to prior years. Sexual assault cases presenting for care dropped by 53.49% and physical assault cases dropped by 48.45%. While the specialized Sexual Assault and Domestic Violence Program patient volume decreased by half, the proportional composition of the presenting patients was similar. The majority of cases occurred among female patients (88.57%), 51.43% were assaulted by an intimate partner, and the most common location of assault was the assailant’s home (40.00%) or the patient’s home (31.43%). This study adds important information to limited evidence base on the impact of the COVID-19 pandemic on care-seeking for sexual assault and domestic violence in EDs.

The observed changes in ED admissions for sexual assault and domestic violence are likely due to a combination of factors including the change in the prevalence of violence during the pandemic (which is hypothesized to be increasing), changes in the severity of events requiring urgent care (which is unknown), fear of contracting COVID-19, and avoiding health care establishments (which is well documented) [25–27, 37, 38]. More complicated factors to consider are being isolated with a violent or controlling partner and unable to seek care, or fewer social interactions that present opportunities for assaults from non-intimate partners (e.g., unknown assailants, acquaintances). While not statistically significant, there was a 10% increase in the proportion of cases presenting via ambulance during COVID-19, supporting findings from other studies that documented increased injury severity among cases that present for care during COVID-19 [11].

Within our sample, the most common assailant was an intimate partner (51.43%). However, there were still cases of assault from unknown assailants (28.57%) and multiple assailants (< 5%). While the number of cases was small, there was a statistically significant increase in the proportion of cases reporting psychological or emotional abuse, from 11.69% (8 cases) in 2018 to 28.57% (9 cases) in 2020. This finding is similar to an online survey of 2441 respondents conducted in the USA that found that 18% of respondents had experienced intimate partner violence since COVID-19, with the most common form being emotional or psychological abuse [39]. Emotional or psychological abuse is priority issues in child maltreatment cases; however, it is often a neglected component of adult domestic violence and sexual assault cases [40], which often focuses on physical trauma or circumstances that meet criminal thresholds [41].

Among the patients that were admitted, the same clinical and forensic care was provided and uptake patterns were similar between years. This is an encouraging finding as it illustrates that the COVID-19 pandemic did not influence care provision among presenting patients, despite the drastic disruption to standard care. While not statistically significant, there was a 16.80% increase in the proportion of patients who released an evidence kit to police, and this is an interesting trend that may reflect patterns of more severe cases presenting for care. In previous studies, we have identified that approximately two thirds of eligible case complete a Sexual Assault Evidence Kit, and one third release it to police [34]. While there are many factors that contribute to the decision to complete evidence collection, it is important to ensure that survivors are fully informed and supported to move forward with legal action if they desire even under the current COVID-19 pandemic conditions.

To account for the potential confounding effects of seasonality, we compared the COVID-19 time frame (March to May) in 2020 and 2018. Previously, we conducted a 15-year population-based analysis and documented that ED sexual assault cases are highest in the summer, decrease through fall and winter, and begin to rise in early spring [42]. Despite the previously identified seasonal increase in the spring, this study documents a decrease in cases presenting for care. Of note, there was a statistically significant increase in the proportion of assaults that occurred outdoors (from 5.19% in 2018 to 22.86% in 2020). This was an unexpected finding as the temperatures in Ottawa between March and May 2020 ranged from − 4 to 10 °C (25 to 50 °F) [43] and outdoor assaults tend to occur more frequently in warmer months. However, the cell sizes are small and there is limited ability to draw conclusions about this association.

Important clinical and public health implications of this research include the need for multi-sectoral collaborations to provide support for the increase in violence occurring in the face of increasing barriers to care because of COVID-19 restrictions. One solution launched by various organizations across the world [44, 45] is the development of a chat-based support line. Chat-based support provides an opportunity for individuals who cannot verbally ask for help to get support for safety planning, emotional support, and connection to services. “Unsafe at Home” was a chat-line developed by Ottawa-based community organization and within the first month received over 300 chatters looking for support, safety planning, and resources [46]. This can also be a useful resource if survivors are hesitant to reach out for support because they were assaulted while not follow physical distancing protocols and may be judged by service organizations.

Relevant research implications include the use of the ED as a reliable source of routinely collected information on sexual assault and domestic violence cases presenting for care. The main bias is that ED cases are typically more severe, or the survivors are willing and able to overcome the social barriers (e.g., stigma, shame) and logistical barriers (e.g., time, money) [11, 12]. An additional challenge is that cases may present for injury care and not disclose that it was a consequence of domestic violence, as has been documented in orthopedic clinics [47]. ED data will underestimate the prevalence of violence in the general population; however, it will be an important source of routinely collected information for measuring the ongoing trends in care-seeking for violence as the society begins to re-open or lock-down again. Within this study, we compared the same time period (March 4 to May 5) in 2018 and 2020 to investigate changes in admissions. An alternative approach could use a Difference-in-Differences study design by including a “pre” period from both years. We considered this approach; however, the two “pre” periods in our study did not meet the parallel trend assumption and we were unable to use this approach.

### Limitations

This study uses ED admissions data from a specialized Sexual Assault and Domestic Violence Program and cannot determine changes in the number of people who experienced violence in the general population, those who sought care elsewhere, or those who did not seek care at all. Additionally, it is possible that not all admitted cases were appropriately triaged to the Sexual Assault and Domestic Violence Program, introducing misclassification bias. There is limited information on sociodemographic factors including race and income. Measurement bias may be present as some of characteristics of the cases are self-reported (e.g., assailant, location, assault characteristics); however, it is comparable between both time periods. This study represents a change in patient volume at a specialized Sexual Assault and Domestic Violence Program at a metropolitan hospital; therefore, it may have higher volumes of assault cases compared to institutions without a specialized program or those in suburban or rural settings. Additionally, the short time frame and limited sample may lead to higher variability in the estimates.

## Conclusion

The COVID-19 pandemic has introduced considerable societal changes that have heightened the risk for violence and decreased opportunities to access care. Within our study, all-cause ED admissions reduced by over 30%, and sexual assault and physical assault cases presenting for care decreased by approximately 50%. Trends in care-seeking and assault patterns will require monitoring over time, and evidence is needed to inform the provision of adequate support for individuals experiencing violence. This is particularly important as cities and countries begin to slowly re-open services and businesses—the negative consequence of quarantine and isolation on violence may become apparent months after the main concern for COVID-19 transmission is reduced.

### Research in context

#### Evidence before this study

We conducted a comprehensive but non-systematic review of PubMed, Embase, various websites of non-governmental organizations, government departments, news sources, and manual checking of reference lists. MeSH terms, keywords, and title/abstract terms used in the search included “COVID-19” and various terms for violence (e.g., domestic violence, physical violence, sexual violence, sexual abuse, intimate partner violence). No date restrictions were applied. The majority of published literature were commentaries or editorials, and there are few studies with empirical findings, particularly within health care settings.

#### Added value of this study

To our knowledge, this is one of the first study investigating sexual assault and domestic violence cases presenting to emergency departments for urgent care during the COVID-19 pandemic, compared to a time-match control period. Compared to pre-COVID-19, total emergency department admissions at The Ottawa Hospital dropped by 1111.2 cases per week (32.9% reduction), and sexual assault and domestic violence cases dropped by 4.66 cases per week (54.6% reduction). There was a significant increase in the proportion of cases with psychological abuse (11.69% vs 28.57%) and assaults occurring outdoors (5.19% vs 22.86%).

#### Implications of all the available evidence

Our results suggest that the decrease in access to services, despite the concerns in increased risks for violence during COVID-19, underscores the need for support for seeking urgent care or alternative care models. Trends in care-seeking and assault patterns will require monitoring over time, and evidence is needed to inform the provision of adequate support for individuals experiencing violence. This is particularly important as cities and countries begin to slowly re-open or go into lock-down again—the negative consequence of quarantine and isolation on violence may become apparent months after the main concern for COVID-19 transmission is reduced.

## Supplementary Information

**Additional file 1: Table S1.** Annual comparison of ED admissions between March 4th and May 4th (*n*=62 days) for 2015, 2016, 2017, 2018, 2020. **Table S2.** Poisson regression modeling the weekly case count ratio in 2020 (COVID-19 period) to time matched 2015, 2016, 2017, 2018 pre-COVID-19 comparison groups.

## Data Availability

With a data sharing agreement, de-identified data, the data dictionary, and ethics protocol are available. The data is held at the Ottawa Hospital Research Institute.
