# Supplementary material for: COVID-19 pandemic and violence: rising risks and decreasing urgent care-seeking for sexual assault and domestic violence survivors
Source: BMC Med. 2021 Feb 5;19:20. doi: 10.1186/s12916-020-01897-z (PMC7861965; doi:10.1186/s12916-020-01897-z)
Supplement: Supplementary file 1 — Additional file 1: Table S1. Annual comparison of ED admissions between March 4th and May 4th (n=62 days) for 2015, 2016, 2017, 2018, 2020. Table S2. Poisson regression modeling the weekly case count ratio in 2020 (COVID-19 period) to time matched 2015, 2016, 2017, 2018 pre-COVID-19 comparison groups. [file 12916_2020_1897_MOESM1_ESM.docx]

**Additional File**

**Table S1. Annual comparison of ED admissions between March 4^th^ and May 4^th^ (n=62 days) for 2015, 2016, 2017, 2018, 2020**

|  | **2015** | **2016** | **2017** | **2018** | **2020** |
| --- | --- | --- | --- | --- | --- |
|  |  |  |  |  |  |
| **ED admissions** |  |  |  |  |  |
| Total ED admissions | 28559 | 28948 | 29817 | 30371 | 20370 |
| Absolute change in weekly ED admissions compared to 2020 | 8189 | 8578 | 9447 | 10001 | (ref) |
| Mean weekly ED admissions | 3173.22 | 3216.44 | 3313.00 | 3374.56 | 2263.33 |
| Mean weekly difference in ED admissions for each year compared to 2020 | 909.89 | 953.11 | 1049.67 | 1111.22 | (ref) |
|  |  |  |  |  |  |
| **Sexual Assault and Domestic Violence Program patients** |  |  |  |  |  |
| Total cases | 60 | 75 | 81 | 77 | 34 |
| Absolute difference in patients for each year compared to 2020 | 25 | 40 | 46 | 42 | (ref) |
| Mean weekly patients | 6.67 | 8.33 | 9.00 | 8.55 | 3.89 |
| Mean weekly change in patients for each year vs 2020 | 2.78 | 4.44 | 5.11 | 4.67 | (ref) |
| Case rate per 10,000 ED admissions | 21.01 | 25.91 | 27.17 | 25.35 | 17.18 |
|  |  |  |  |  |  |
| **Sexual assault** |  |  |  |  |  |
| Total cases | 43 | 38 | 45 | 46 | 20 |
| Absolute difference in cases for each year vs 2020 | 23 | 18 | 25 | 26 | (ref) |
| Mean weekly cases | 4.78 | 4.22 | 5.00 | 5.11 | 2.22 |
| Mean weekly change in cases for each year vs 2020 | 2.56 | 2.00 | 2.78 | 2.89 | (ref) |
| Case rate per 10,000 ED admissions | 15.06 | 13.13 | 15.09 | 15.15 | 9.82 |
|  |  |  |  |  |  |
| **Physical assault** |  |  |  |  |  |
| Total cases | 20 | 26 | 32 | 33 | 17 |
| Absolute change in cases for each year vs 2020 | 3 | 9 | 15 | 16 | (ref) |
| Mean weekly cases | 2.22 | 2.89 | 3.56 | 3.67 | 1.88 |
| Mean weekly difference in cases for each year vs 2020 | 0.33 | 1.00 | 1.67 | 1.78 | (ref) |
| Case rate per 10,000 ED admissions | 7.00 | 8.98 | 10.73 | 10.87 | 8.35 |

**Table S2.** **Poisson regression modelling the weekly case count ratio in 2020 (COVID-19 period) to time matched 2015, 2016, 2017, 2018 pre-COVID-19 comparison groups**

| **Time frame:**  **March 4^th^ to May 5^th^** | **Weekly Case Count Ratio**  **(95% CI)** | **p-value** |
| --- | --- | --- |
| **Sexual Assault and Domestic Violence Program patients** |  |  |
| 2015 | 1.71 (1.23-2.60) | 0.011 |
| 2016 | 2.14 (1.43-3.20) | <0.001 |
| 2017 | 2.31 (1.56-3.44) | <0.001 |
| 2018 | 2.20 (1.47-3.28) | <0.001 |
| 2020^1^ | (ref) |  |
|  |  |  |
| **Sexual assault** |  |  |
| 2015 | 2.15 (1.26-3.65) | 0.005 |
| 2016 | 1.90 (1.11-3.27) | 0.020 |
| 2017 | 2.25 (1.33-3.81) | 0.003 |
| 2018 | 2.30 (1.36-3.89) | 0.002 |
| 2020 | (ref) |  |
|  |  |  |
| **Physical assault** |  |  |
| 2015 | 1.18 (0.616, 2.25) | 0.622 |
| 2016 | 1.52 (0.83, 2.82) | 0.173 |
| 2017 | 1.88 (1.05, 3.39) | 0.035 |
| 2018 | 1.94 (1.08, 3.49) | 0.026 |
| 2020 | (ref) |  |

1. 2020 is the reference year, all estimates indicate an increase in the case count in each year compared to 2020
